# Supplementary material for: Environmental co-benefits and adverse side-effects of alternative power sector decarbonization strategies
Source: Nat Commun. 2019 Nov 19;10:5229. doi: 10.1038/s41467-019-13067-8 (PMC6864079; doi:10.1038/s41467-019-13067-8)
Supplement: Supplementary file 2 — Supplementary Information [file 41467_2019_13067_MOESM2_ESM.pdf]

Supplementary Information

Environmental co-benefits and adverse side-effects of alternative power sector decarbonization strategies

Gunnar Luderer et al.

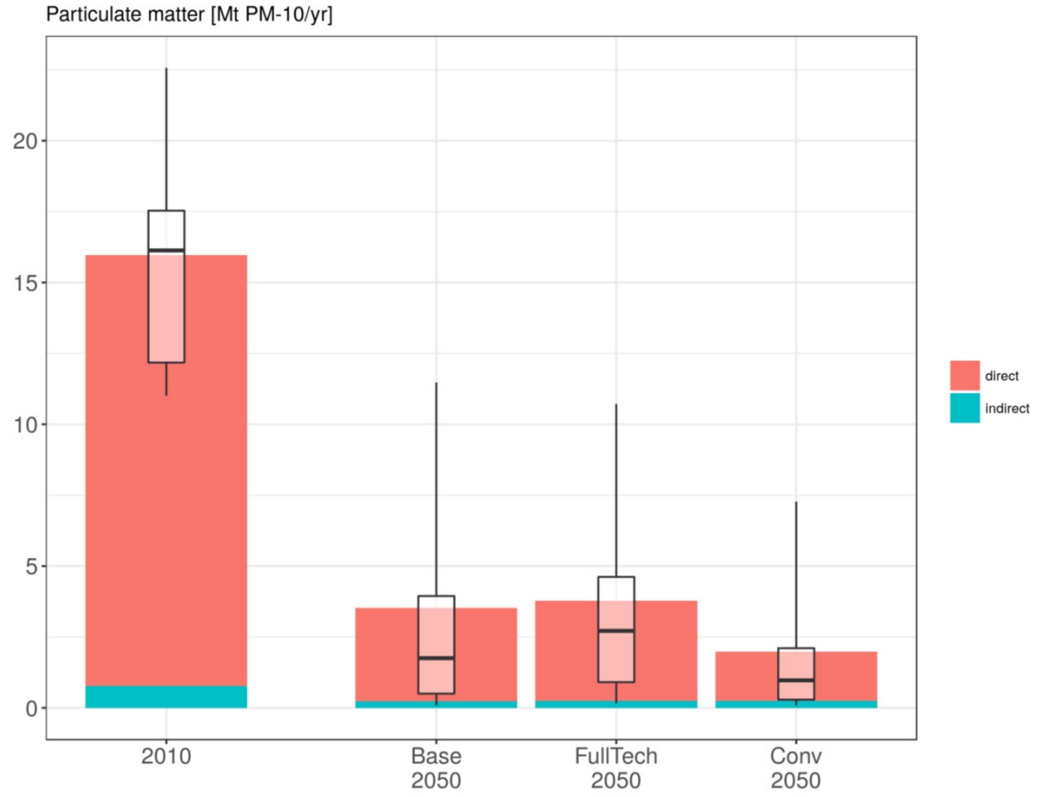

Supplementary Figure 1| Direct and indirect particulate matter emissions. Relative contribution of direct emissions (from combustion power technologies) and indirect emissions (from upstream activities) to particulate matter formation.

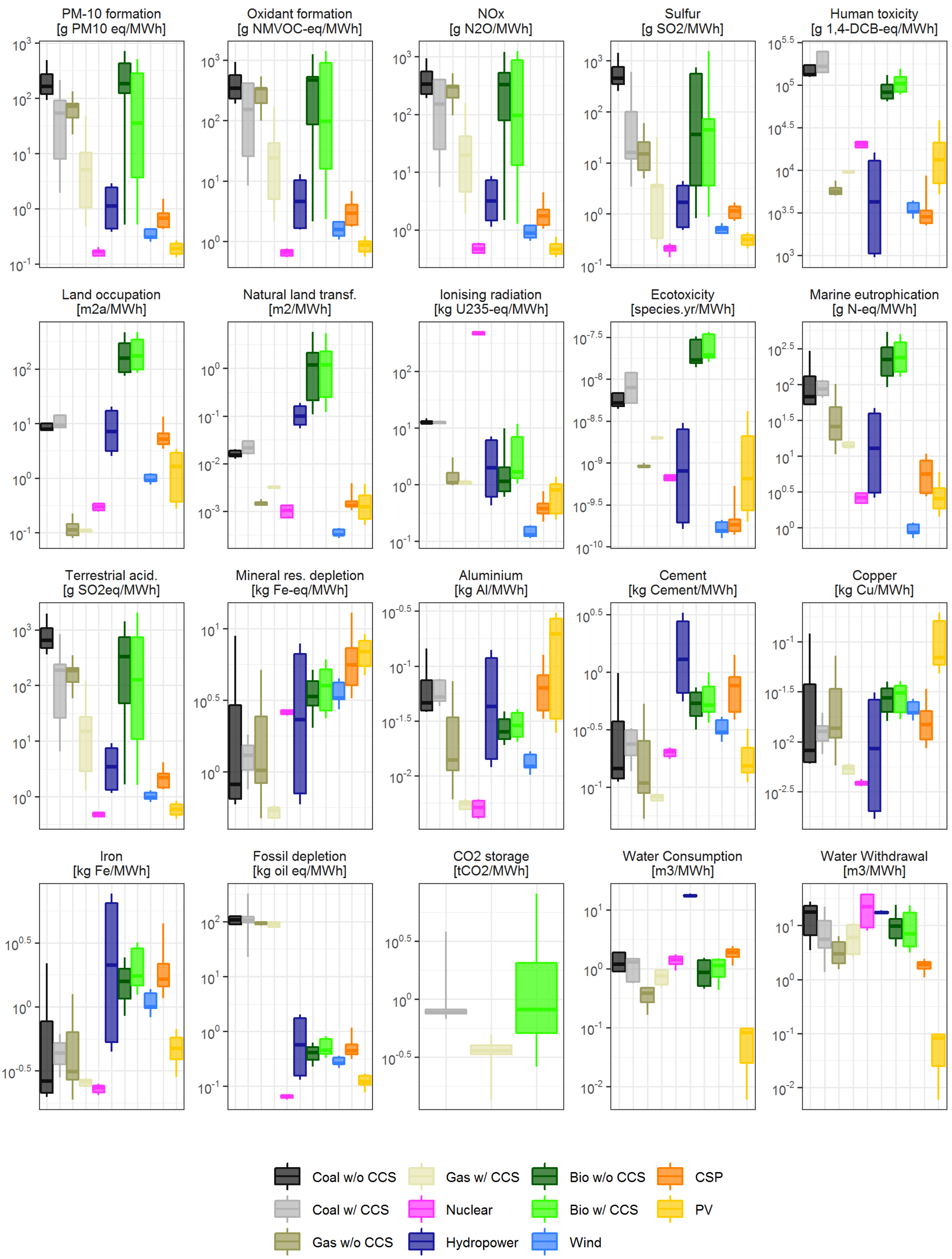

Supplementary Figure 2| Technology specific environmental impacts. Per unit life-cycle environmental impacts of electricity technologies for the *FullTech* scenario and the year 2050. Boxplots indicate median and interquartile ranges across technology variants and participating integrated assessment models, whiskers 10th-90th percentile ranges.

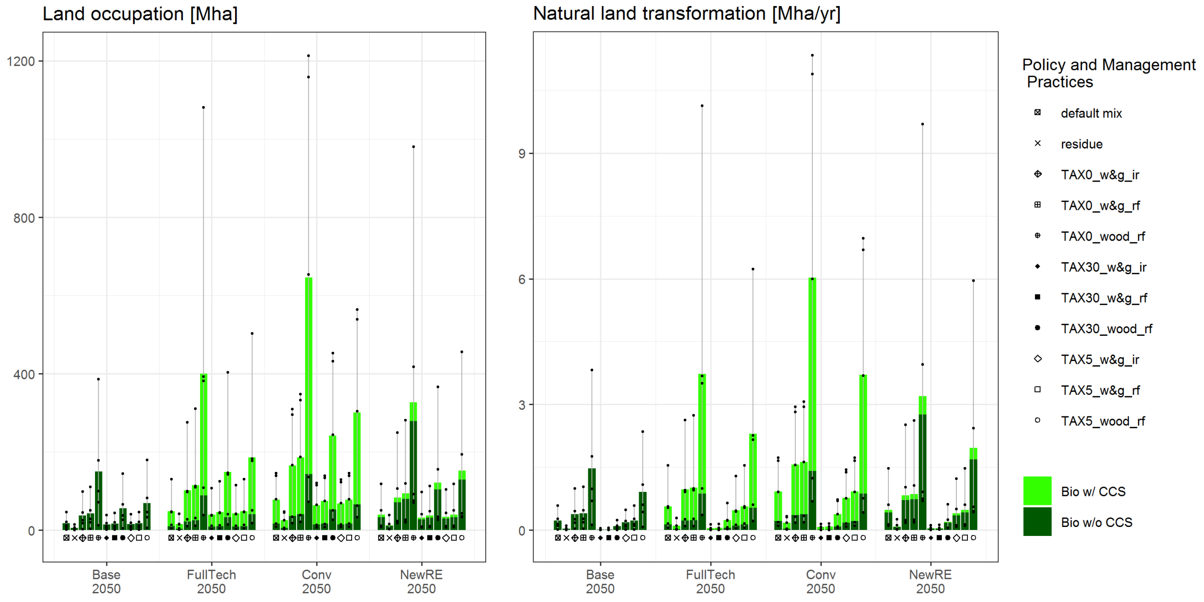

Supplementary Figure 3| Uncertainties in land occupation and natural land transformation impacts from bioenergy. Individual bars show impacts attributed to bioenergy with and without bioenergy, for various assumptions regarding land use policies (carbon taxes of 0, 5, 30 $/tCO2 applied in the land sector from 2030, increasing at 5 %p.a., in the “TAX30”, “TAX5” and “TAX0”) and management practices (only woody feedstocks “wood” or grassy and woody feedstocks “w&g”; irrigated “ir” or rainfed-only “rf” bioenergy cultivation). Connected dots give results for individual IAM scenario realizations. Residues have comparatively low impacts. As a default, we assumed 30% residues and 70% purpose grown biomass under TAX5_w&g_rf (weak carbon pricing of land systems, grassy and woody biomass, rainfed-only). We find that these policies and practices have a very strong impact on environmental impacts of bioenergy. Overall, variations in impacts due to uncertainty in bioenergy demands across models as well as policy and management practices exceed variations across technology scenarios.

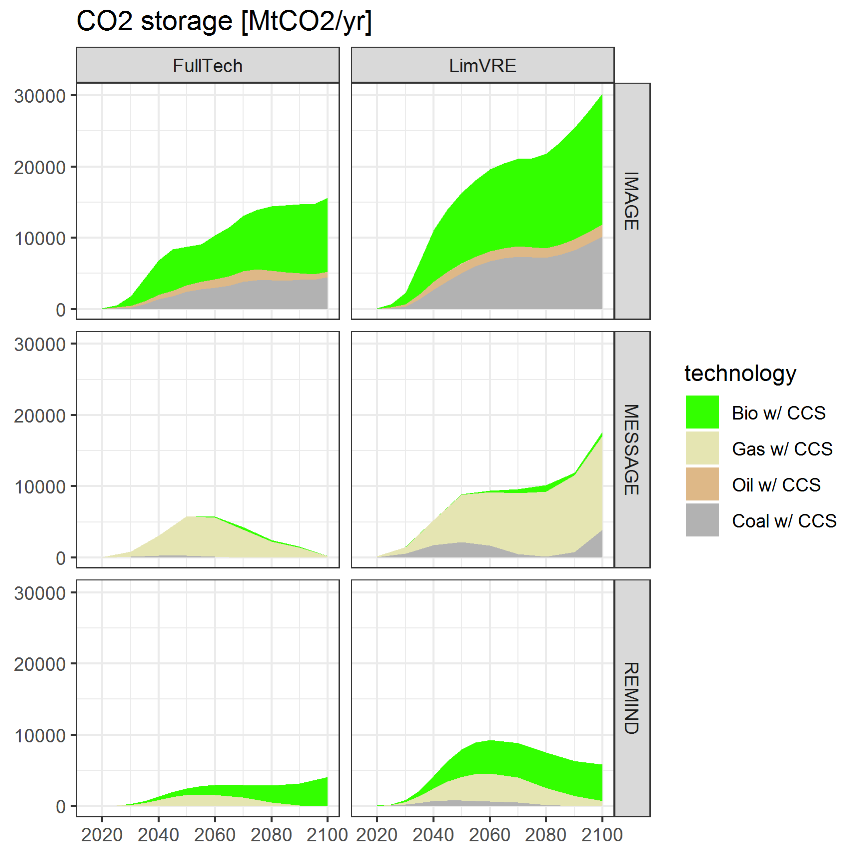

Supplementary Figure 4| CO_2 storage by power-sector CCS technologies through 2100. CCS storage requirements of the power sector increase after 2050 in the majority of model-scenario combinations, mostly due to increased BECCS deployment. At the same time, the power sector competes with other energy conversion pathways for limited storage capacities, resulting in decreasing power sector CO_2 storage in the MESSAGE-FullTech and REMIND-LimVRE scenarios. POLES and GCAM did not report scenario results beyond 2050.

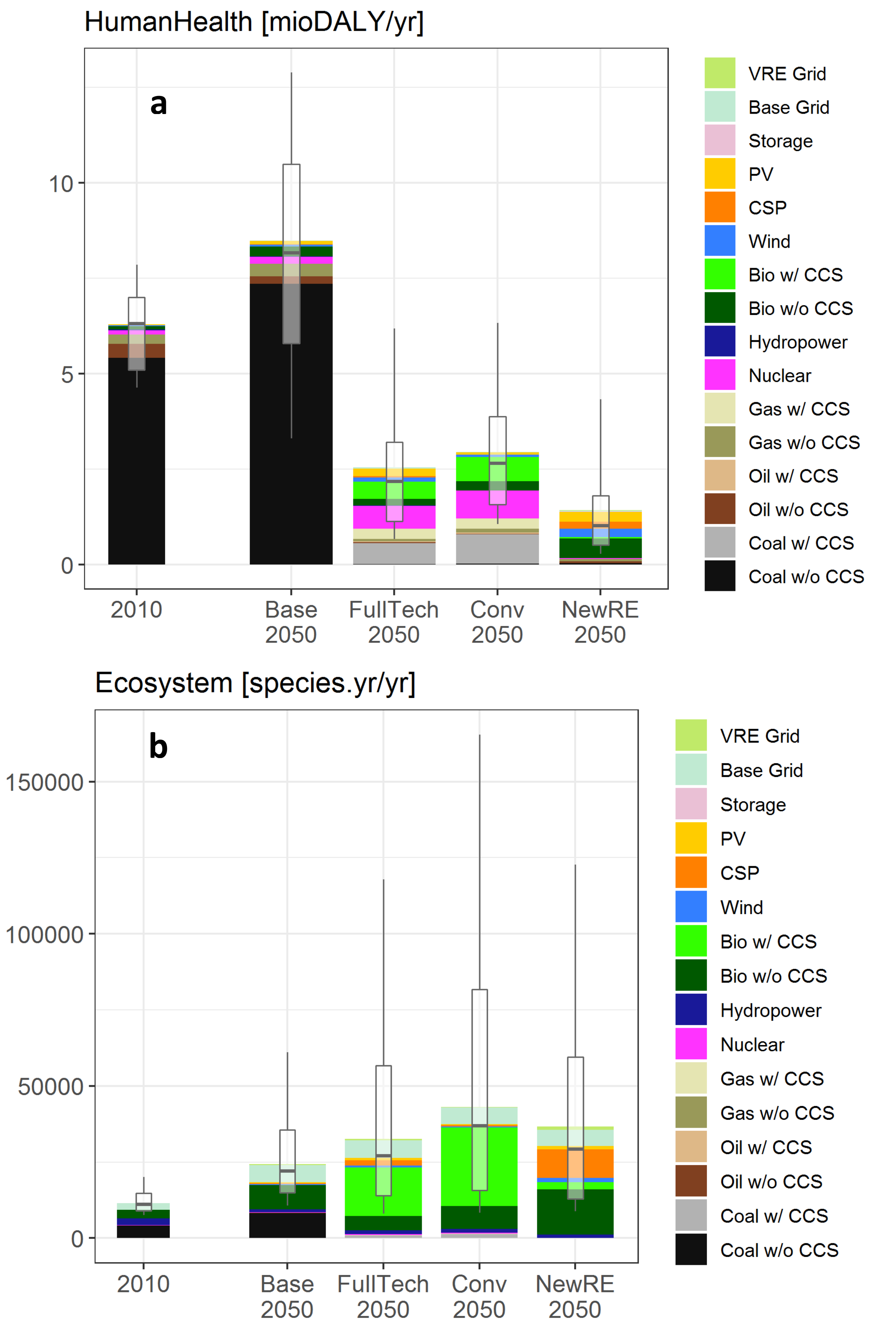

Supplementary Figure 5| Endpoint impacts. Aggregate endpoint impacts on (a) human health and (b) ecosystem damages broken down by technology. . Boxplots indicate median and interquartile ranges across technology variants and participating integrated assessment models, whiskers 10th-90th percentile ranges.
