# Supplementary material for: Environmental co-benefits and adverse side-effects of alternative power sector decarbonization strategies
Source: Nat Commun. 2019 Nov 19;10:5229. doi: 10.1038/s41467-019-13067-8 (PMC6864079; doi:10.1038/s41467-019-13067-8)
Supplement: Supplementary file 3 — Description of Additional Supplementary Files [file 41467_2019_13067_MOESM3_ESM.pdf]

Description of Additional Supplementary Files

File Name: Supplementary Data 1
Description: Data from energy transformation pathways as calculated by the IAMs.

File Name: Supplementary Data 2
Description: Result data on scenario and model specific environmental impacts.

File Name: Supplementary Data 3
Description: Technology-specific environmental impact coefficients aggregated to the midpoint and endpoint levels, based on the THEMIS model.

File Name: Supplementary Data 4
Description: Coefficients of bioenergy-related or bioenergy-induced cropland, land transformation, land-use-change emissions, non-CO2-GHG emissions, water withdrawal and fertilizer use derived from the MAgPIE model.
